# Incomplete Vaccination Among Children With Special Health Care Needs in Zhejiang, China: Analysis of Retrospective Data

**DOI:** 10.3389/fped.2019.00173

**Published:** 2019-05-01

**Authors:** Mingyan Li, Chai Ji, Bin Wang, Dan Yao, Xia Wang, Yan Zeng, Jie Shao

**Affiliations:** Department of Child Health Care, Children's Hospital Zhejiang University School of Medicine, Hangzhou, China

**Keywords:** vaccination, contraindication, correlative factors, adverse events following immunization, children with special care needs

## Abstract

**Objective:** There is a lack of data relating to vaccination of children with special health care needs (CSHCN) and its influencing factors in China. We investigated the disease spectrum of CSHCN at the Vaccination Consultation Clinic in Zhejiang province as well as the underlying factors of vaccination recommendations of these children.

**Methods:** In this study, we retrospectively analyzed the data of 4,525 CSHCN, who visited to our clinic for a vaccination consultation from January 1, 2016 to May 30, 2018. Descriptive data were presented as mean ± standard deviation (SD) and percentages. Multivariate analysis was performed with non-conditional bivariate logistic regression to identify the underlying factors of vaccination recommendations. Subsequent information regarding the following vaccination and the occurrence of AEFI were also collected and analyzed.

**Results:** The main diseases consulted were those relating to the circulatory and nervous systems as well as neonatal diseases. The distribution of diseases varied by age: 53.6% infants under 12 months were counseled for circulatory system diseases, while 44.6% children aged 12~24 months and 54.7% children over 25 months were counseled for nervous system diseases. According to the evaluation reports issued by the consultation clinic, 75.0% of CSHCN were recommended to be vaccinated normally, 21.2% were recommended to defer specific vaccination, while only 3.8% were recommended to defer all vaccinations. In logistic regression analysis, age, history of adverse events following immunization (AEFI) and the number of diseases combined were all strong correlative factors for vaccination recommendations. Children who were aged over 25-month-old (OR = 1.34, 95%CI: 1.11–1.61) or had a history of AEFI (OR = 3.77, 95%CI: 2.83~5.01) or those who had numerous diseases combined (OR = 2.00, 95%CI: 1.46~2.75) tended to have a higher rate of deferred vaccination recommendation. Among those CSHCN who received nationally-recommended vaccines, the estimated AEFI rate was 24.29/100 000. No uncommon or rare serious adverse reactions were detected.

**Conclusion:** Age, history of AEFI, and the number of diseases combined were important factors that affected the vaccination recommendations of CSHCN. Most CSHCN can be safely vaccinated according to the nationally-recommended schedule.

## Introduction

Vaccination is one of the most cost-effective interventions of preventing infectious disease and reducing the burden of disease (1). It makes up a significant component of the prevention of comprehensive preventive and primary care services for all children, including those with special medical conditions.

Children with special health care needs (CSHCN) are those who have or are at increased risk for physical, developmental, behavioral, or emotional disorders (2). They always require special health services beyond the basic healthcare that is provided for the general public. It is common for vaccination providers to encounter CSHCN who suffering from various diseases, like congenital heart disease (CHD), seizure, thrombocytopenic purpura or various allergic diseases.

The vaccination coverage varies significantly among CSHCN in different countries. For example, from the national survey in the United States, it is reported that CSHCN have similar vaccination rates as normally developing children (3); while in Brazil, vaccination rates are low (4). The underlying factors of incomplete vaccination are complicated. A previous literature review stated that demographic characteristics such as age, gender, insurance status are correlates of receiving recommended vaccines (5). Some other studies focusing on the impact of some socioeconomic factors on vaccination and indicated that the outreach of medical support programs targeted to CSHCN, family's socioeconomic status and parental educational level are all related to the vaccination coverage (3, 4, 6).

In China, the vaccination coverage in recent years has been maintained at an optimal level for the general population (7), but the immunization status in CSHCN remains unknown. In accordance with “Chinese Immunization Program for Children Immunization Procedures and Instructions (2016),” children under the age of 6 (except for neonates, who receive hepatitis B vaccine and bacillus Calmette-Guérin vaccine in the hospitals where they were born) receive vaccines at local community health centers. Owing to the incomplete understanding of contraindications of vaccination against CSHCN, providers in community health centers often feel hesitant to give vaccines to CSHCN. And for the sake of safety, they tend to overestimate the contraindications and refuse to immunize CSHCN on time. As a result, many CSHCN eligible for vaccinations delayed or even missed the opportunity for immunization. This not only affects the coverage or timeliness of vaccination, but also increases the risk of infection of the vaccine preventable diseases for CSHCN.

To improve the safety of vaccination as well as to increase the vaccination coverage among CHSCN in Zhejiang, China, Health Commission of Zhejiang Province set up the Vaccination Consultation Clinic in our hospital in 2016. Specialists of the Consultation Clinic provide the vaccination recommendation to the group of children, who were denied vaccination at community health centers.

We conducted the present study, a retrospectively analysis of the data of CSHCN who visited our Vaccination Consultation Clinic, in order to gain insight into the immunization status in CSHCN in Zhejiang province and to identify correlative factors that affect vaccination recommendations. The result of the study can contribute to the existing literature on the vaccination of CSHCN and help us to explore new strategies of improving clinicians' and providers' understanding of the contraindications and precautions of vaccination in China.

## Methods

### Study Design and Measures

The Vaccination Consultation Clinic was established in Children's hospital Zhejiang University School of Medicine in 2016. Pediatricians who were systematically trained in vaccinology and public health were responsible for the consulting work.

CSHCN was defined as children who meet at least one of the following criteria resulting from a medical condition that lasted, or was expected to last ≥12 months: (1) ongoing need of prescription medications; (2) on going need of health care services above the average for a child the same age; (3) on going need of specialized treatments; (4) limitation in activities that most children the same age can perform; (5) presence of ongoing behavioral or developmental conditions requiring treatment or counseling (8).

In this study, CSHCN who visited the clinic from January 1, 2016 to May 30, 2018 were enrolled. Medical records of treatments, disease history, family history, allergic history, vaccination status and AEFI history were collected from parents during the interview. Relevant laboratory tests were conducted if seen to be necessary.

Children were evaluated according to their medical records and lab results. Specialists of the Consultation Clinic have a meeting once a week to conduct discussions on the vaccination plans for each CSHCN. Referring to “Chinese Immunization Program for Children Immunization Procedures and Instructions (2016), “Chinese Pharmacopeia” and “the General Best Practice Guidelines for Immunization (2016) (9, 10), and combined with children's medical records and lab experiment results, consensus on vaccination recommendations are issued then.

The vaccination recommendation included three options: (1) defer all vaccinations, which meant children should postpone all vaccinations due to the moderate to severe illness, or because they had an unstable chronic diseases; (2) defer specific vaccination, which meant children should postpone one or more specific vaccines due to their current disease, treatment or history of AEFI; (3) normal vaccination, which meant children could receive all vaccines according to the nationally-recommended schedule.

Providers in community health centers carried out the vaccination in accordance with the vaccination recommendations. Local CDC monitored and recorded vaccination and uncommon/rare serious AEFI of those CSHCN. The following vaccination and the occurrence of AEFI were collected by telephone interview from parents at an interval of 6 months after the consultation.

### Statistical Analysis

The data were extracted from the hospital information management system. All data were managed and analyzed by SPSS 20.0. Descriptive data were presented as mean ± standard deviation (SD) and percentages. The vaccination recommendations were categorized into two levels: The first level was “normal vaccination,” and the other was “delayed vaccination,” which combined “defer all vaccinations” and “defer specific vaccination.” Multivariate analysis was performed with non-conditional bivariate logistic regression to determine significant independent variables for predicting vaccination recommendations. All predictor variables were entered in the equation as dummy variables. The alpha level of significance for bivariate logistic analysis was set at 0.05 for a two-tailed test.

### Ethics Statement

The study was approved by the ethics committee of the Children's Hospital Zhejiang University School of Medicine. The need for informed consent was waived by the Ethics Committee of the Children's Hospital Zhejiang University School of Medicine.

## Results

### Sample Characteristics

A total of 4,525 children were included in our analysis. Participants ranged in age from 0 month to 15 years old; the mean age was 14.2 months (SD = 19.1). Almost half (50.7%) of the participants were under 6 months old; among them, 56.7% were male; 27.8% had a history of food or drug allergies; 4.7% had a history of AEFI; 20.9% had a family history of related disease; 61.2% were consulted for only one disease shown in Table 1.

**Table 1:** Sample characteristics (*N* = 4,525).

	***N***	**%**
**AGE (MONTHS)**		
0–6	2,294	50.7
7–12	753	16.6
13–24	639	14.1
≥25	839	18.5
**GENDER**		
Male	2,566	56.7
Female	1,959	43.3
**HISTORY OF ALLERGY**		
Yes	1,256	27.8
No	3,269	72.2
**HISTORY OF AEFI**		
Yes	213	4.7
No	4,312	95.3
**FAMILY HISTORY**		
Yes	946	20.9
No	3,579	79.1
**NUMBER OF COMBINED DISEASES**		
1	2,769	61.2
2	1,159	25.6
3	402	8.9
4	195	4.3

### Disease Spectrum of CSHCN

**Figure 1:**
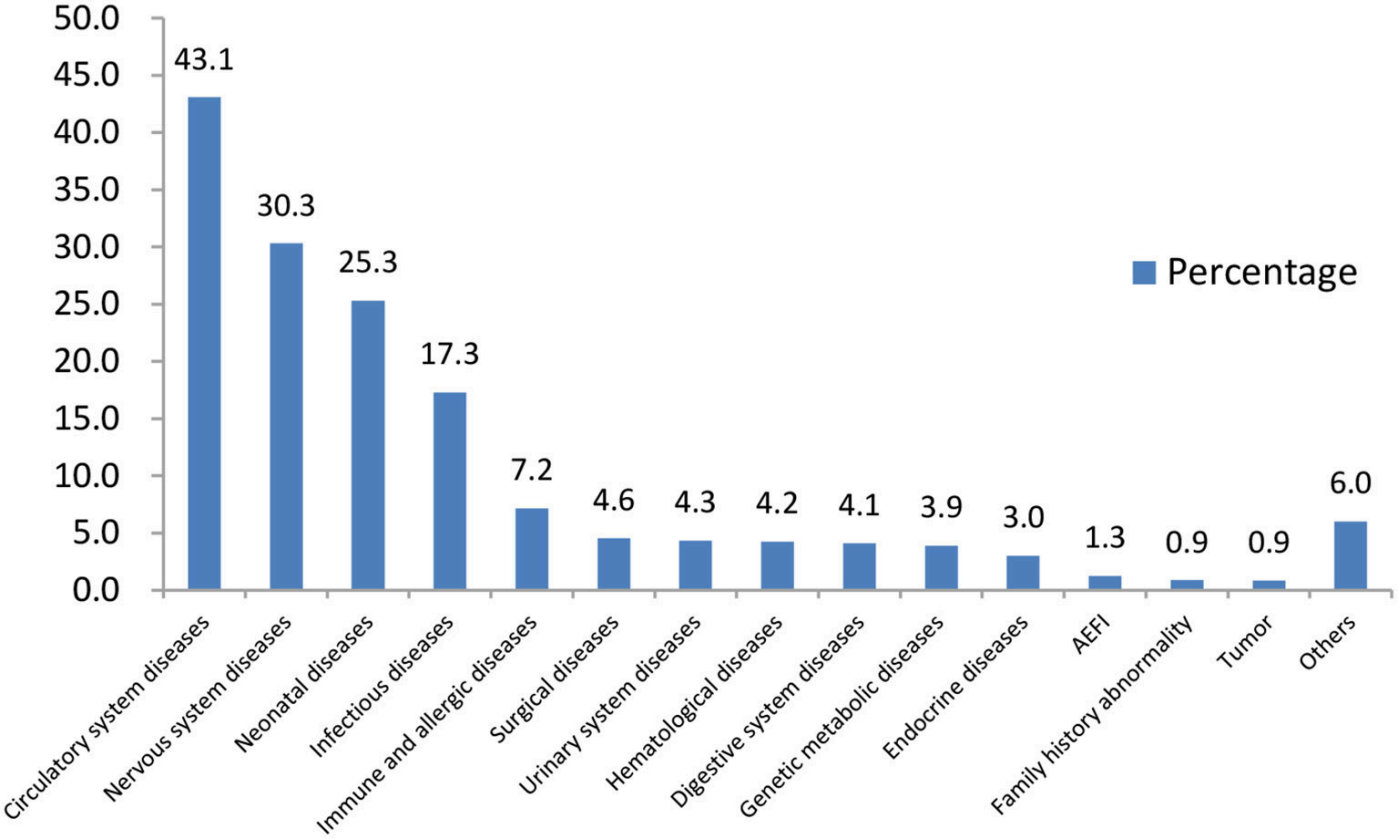
Characteristics of disease spectrum.

The main diseases of those CSHCN for consultation were circulatory system diseases (43.1%), nervous system diseases (30.3%) and neonatal diseases (25.3%). The proportions of children with a history of AEFI and a family history of related disease were 1.3 and 0.9%, respectively (Figure 1).

The distribution of diseases was different between CSHCN that were of different ages. Infants with 0~6 months and 7~12 months visiting the clinic were mostly for circulatory system diseases, especially the CHD (96.4 vs. 95.4%); children aged 12~24 months and over 25 months old mostly consulted for nervous system diseases, among which febrile convulsion (37.6 vs. 41.4%), developmental delay(20.7 vs. 15.1%) and epilepsy (16.5 vs. 20.7%) were the top 3 causes.

### Factors Associated With Vaccination Recommendations

According to the evaluation reports issued by the consultation clinic, 3,395(75.0%) of the evaluated CSHCN were recommended to receive vaccines normally according to the nationally-recommended schedule, and 958(21.2%) were recommended to defer specific vaccination, while only 171(3.8%) were recommended to defer all vaccinations.

Of the CSHCN with the recommendation of “defer specific vaccination” or “defer all vaccination”, most had a history of AEFI (57.9, 3.5%), tumors (41.0, 5.1%), infectious disease (37.0, 2.8%), hematological disease (35.9, 7.3%) or immune, and allergic disease (33.3, 5.6%) (Table 2).

**Table 2:** Vaccination recommendations for different diseases.

	**Defer all vaccination**		**Defer specific vaccination**		**Normal vaccination**		**Total**	
	***n***	**%**	***n***	**%**	***n***	**%**	***n***	**%**
Circulation system diseases	43	2.2	305	15.7	1601	82.1	1949	43.1
Nervous system diseases	90	6.6	240	17.5	1041	75.9	1371	30.3
Neonatal diseases	20	1.8	273	23.8	852	74.4	1145	25.3
Infectious diseases	22	2.8	289	37.0	471	60.2	782	17.3
Immune and allergic diseases	18	5.6	108	33.3	198	61.1	324	7.2
Surgical diseases	6	2.9	53	25.7	147	71.4	206	6.0
Urinary system diseases	5	2.6	36	18.4	155	79.1	196	4.3
Hematological system diseases	14	7.3	69	35.9	109	56.8	192	4.2
Digestive system diseases	10	5.4	47	25.1	130	69.5	187	4.1
Genetic metabolic diseases	6	3.4	49	27.7	122	68.9	177	3.9
Endocrine diseases	5	3.7	24	17.5	108	78.8	137	3.0
AEFI	2	3.5	33	57.9	22	38.6	57	1.3
Abnormal family history	2	5.0	6	15.0	32	80.0	40	0.9
Tumor	2	5.1	16	41.0	21	53.9	39	0.9
Others	8	2.9	65	23.9	199	73.2	272	6.0

Binominal regression analysis indicated that age, history of AEFI and the number of combined diseases were important factors of vaccination recommendations (Table 3). Subjects older than 25 months were more likely to receive the vaccination recommendation of “delayed vaccination” than those aged under 6 months (OR = 1.34, 95%CI 1.11~1.61); children who had a history of AEFI in previous immunization were three times more likely to get a “delayed vaccination” recommendation (OR = 3.77, 95%CI: 2.83~5.01). Children with more combined diseases were more likely to be ascribed into the group of “delayed vaccination.” Therefore, age, AEFI history, and disease history are important factors relating to the vaccination recommendations.

**Table 3:** Odds ratios with their respective confidence intervals (95% CI) of risk factors for vaccination among CSHCN.

**Predictor**		**β**	**S.E**	**Wald**	***P***	**OR**	**95%CI**
Gender	Male		–				
	Female	−0.042	0.071	0.345	0.557	0.959	0.835–1.102
Age(months)	0–6	–	–				
	7–12	0.191	0.098	3.770	0.052	1.211	0.998–1.468
	13–24	0.208	0.106	3.834	0.050	1.232	1.000–1.517
	≥25	0.289	0.097	8.951	0.003^*^	1.336	1.105–1.614
Family history	No		–				
	Yes	0.009	0.086	0.012	0.914	1.009	0.852–1.195
Allergic history	No	–	–				
	Yes	0.104	0.078	1.804	0.179	1.110	0.953–1.292
AEFI	No	–	–				
	Yes	1.326	0.146	82.653	<0.001^**^	3.765	2.829–5.011
Number of diseases combined	1	–					
	2	0.364	0.082	19.690	<0.001^**^	1.439	1.226–1.691
	3	0.557	0.121	21.139	<0.001^**^	1.746	1.377–2.215
	4	0.694	0.162	18.332	<0.001^**^	2.002	1.457–2.752

*OR, Odds ratios; CI, confidence intervals. Table shows results of Non-conditional bivariate logistic regression model*.

* P < 0.01;

** *P < 0.001*.

### Occurrence of AEFI

In our study, 3,395 enrolled CSHCN were given vaccines normally according to the nationally-recommended schedule after consultation, among which, 2,976 continued follow-up, while 417(12.29%) children dropped-out for a variety of reasons, such as changing telephone number, unwilling to give information or forgetting specific information. Among the 2,976 valid cases, a total of 20,587 vaccine doses were administered and only five cases experienced adverse reactions (24.29/100,000), two experienced low-grade fevers, one experienced a rash and two presented with local redness at the injection site. These reactions were mild and self-limiting, and did not require medical treatment. No uncommon or rare serious adverse reactions were reported.

## Discussion

To our knowledge, this was the first study on the vaccination and its correlative factors among CSHCN in Zhejiang, China. The main diseases consulted were circulatory and nervous system diseases as well as neonatal diseases. Age, history of AEFI and the number of combined disease were correlative factors of vaccination recommendations. Most CSHCN who visited the consultation clinic were safely vaccinated according to the nationally-recommended schedule after consultation.

In the present study, we found that nearly 50% of the children visiting the consultation clinic were under 6 months old. The probable reason for this was that most vaccinations were scheduled under 12 months of age, with 1~3 doses per month in average (10). Clinicians and parents usually pay more attention to diseases detected during the prenatal examinations and neonatal screenings. Parents are very cautious of the adverse effects of vaccinations if the infant has a health problem. Therefore, there was a higher demand for vaccination consultations for young infants.

In this CSHCN group, circulatory system diseases, especially CHD, were the most frequently consulted diseases for infants under 12 months old. Routine physical examinations during the first year of life and the use of ultrasound made the diagnosis of CHD sensitive and accurate in younger infants. It was estimated that the incidence of CHD at birth was about 0.8% (11). About a quarter of infants born with a heart defect had severe CHD (12). Many vaccines in China, such as BCG, JEV-L, MCV 4 and MPV-A, “heart disease” is clearly classified as a contraindication in the instructions. However, the definition of “heart disease” is unclear, which made many providers hesitate to give vaccines to children who suffer from CHD. Therefore, CHD has become the main cause for consultation in our clinic. However, the mechanism of the effect of CHD on vaccination remains up till now unclear. In some developed countries, specialists even consider children with CHD as the priority population for vaccination (13). Similarly, domestic experts have also gradually reached a consensus that CHD is not the permanent contraindication for vaccinations (14). Therefore, providers need to conduct a specific analysis based on the type of CHD rather than blindly delaying or suspending the vaccination of such groups of children.

The second cause of consultation was neurological diseases, most of which was convulsions. Convulsions were clinically unpredictable. In some cases, children experienced febrile convulsion or seizures for a short period of time after receiving the vaccine (15, 16), which made many clinicians, providers and parents refuse or delay the consequent vaccinations. In fact, so far, there has been no evidence of a direct causal relationship between vaccination and convulsions. In a population-based cohort study, Verbeek et al. analyzed the data of 990 children who developed seizures after vaccination and found that the underlying cause of seizures were genetic or structural abnormalities, rather than vaccines (17). In addition, Lateef et al. analyzed 165 cases of vaccine-related brain injury, and found that many children had a history of neurological abnormalities prior to receiving vaccines (18). Therefore, in most cases, fever or other discomforts caused by vaccines can induce convulsions, but vaccination itself is not the root cause. As vaccination providers, they should have more concerns on some potential diseases in children, instead of simply linking vaccine to convulsions (19, 20).

According to the vaccination evaluation, the majority of CSHCN enrolled can receive vaccines following the nationally-recommended schedule. However, their vaccine schedules were usually delayed before they visited the consultation clinic. We inferred that the possible reasons are as follows: Firstly, it was due to the unclear description for contraindications in the instructions of some vaccines, as well as the lack of evidence regarding the safety and efficacy for CSHCN. Secondly, most vaccination providers at community health centers were majoring in Public Health rather than Clinical Medicine. They do not receive systematic training for vaccinology and lack clinical experience, which prevents them from making the correct judgment for the precautions and contraindications of vaccination for CSHCN (21–24). In addition, due to the negative reports about vaccines and the lack of knowledge regarding the risk of vaccine preventable diseases, parents usually have many concerns for the use of vaccines (5). Finally, for clinical specialists, they have more concerns regarding AEFI if the child are suffering a chronic disease, such as seizures or suppressed immunologic system responses (25). For the reasons mentioned above, CSHCN in our country are usually regarded as a group who are unfit to be vaccinated, which leads to their failure of receiving the vaccines on time.

In order to explore new strategies of improving the vaccination coverage of CSHCN, it was important to identify the main correlative factors that affect vaccination of this population. In the United States, demographic characteristics such as age, gender, insurance status are considered to be associated with vaccination coverage in youths with special health care (5, 26). In our study, we found that gender, family history of related diseases and history of mild or non-vaccine-related allergies were not strong factors influencing vaccination recommendations, which was consistent with the recommendation from “the guidelines for immunization of ACIP” and “Chinese Pharmacopeia”; nevertheless, we found that age, history of AEFI and the number of combined diseases were indicated as strong factors influencing vaccination recommendations. As a child grows up, the incidence of some diseases, like seizure, thrombocytopenic purpura, Kawasaki disease and tumors may increase, which explains why age and combined diseases are important factors in deferred vaccination. The history of AEFI is another important factor because the identified vaccine-related uncommon AEFI was a contraindication of the subsequent doses. As such, it might be important to investigate all AEFIs and to identify the underlying relationship between vaccination and AEFI, which could help the providers to evaluate the potential risk of the current vaccinations more accurately.

In this study, the incidence of AEFI among the follow-up children was low (24.29/100,000) and no uncommon or rare serious adverse reactions were observed. It was consistent with the results from the similar analyses at national level (27). Our results indicated that the majority of CSHCN receiving the vaccines as recommended were safe, which would enhance the confidence on the vaccine safety among CSHCN.

Altogether, the results of our study increase the awareness of primary healthcare workers/providers on vaccination for CSHCN. It is urgent to identify the barriers of vaccination from patients, families, providers and vaccination delivery systems. Innovative strategies should be implemented to improve the vaccination coverage and reduce missed opportunities for CSHCN, such as strengthening the training of providers in the community health center to help them to fully understand the contraindications and precautions of vaccination and to increase parents' awareness of vaccines.

The limitation of the present study is mainly related to its reliance on parent-reported immunization status and the occurrence of AEFI. Specifically, some parents could not accurately recall the mild side effects of vaccination. Furthermore, our vaccination evaluation objects mainly come from Zhejiang province, which did not represent the situation of CSHCN in the entire country. Future longitudinal multicenter studies with larger sample sizes are needed to monitor vaccination coverage, efficacy and the incidence of AEFI in such population. It is hoped that the vaccination rate of CSHCN in Zhejiang, China will be improved through further data accumulation and research.

## Conclusion

The majority of CSHCN can be vaccinated according to nationally- recommended schedule, especially the children suffering from CHD, febrile convulsion and developmental delay. It is very necessary to conduct intensive training on vaccinology and public health among clinicians and vaccination providers, so they can have a better understanding of the contraindications and precautions of vaccination, and provide correct and appropriate advice to CSHCN. Additionally, children with a history of AEFI or multiple diseases should be screened more carefully before receiving vaccines.

## Ethics Statement

The study was approved by the ethics committee of the Children's Hospital Zhejiang University School of Medicine. The need for informed consent was waived by the Ethics Committee of the Children's Hospital Zhejiang University School of Medicine.

## Author Contributions

CJ designed the research. DY, XW, and YZ interviewed respondents and entered data. ML and BW analyzed the data and performed the statistical analyses. ML, CJ, and JS wrote and revised the manuscript. All authors approved the final content of the manuscript.

### Conflict of Interest Statement

The authors declare that the research was conducted in the absence of any commercial or financial relationships that could be construed as a potential conflict of interest.
